# Supplementary material for: Effectiveness and Safety of Ayurvedic Medicines in Type 2 Diabetes Mellitus Management: A Systematic Review and Meta-Analysis
Source: Front Pharmacol. 2022 Jun 8;13:821810. doi: 10.3389/fphar.2022.821810 (PMC9213670; doi:10.3389/fphar.2022.821810)
Supplement: Supplementary file 1 [file DataSheet1.zip › Supplementary Material/Forest and Funnel Plots/Momordica charantia/Subgroup analyses - country/Momordica charantia_FBG_country subgroup analysis.pdf]

Momordica charantia - FBG - country subgroup analysis

Study	Intervention N	Intervention Mean	Intervention SD	Comparator N	Comparator Mean	Comparator SD	Mean Diff. with 95% CI	Weight (%)
**South Asia**								
Suthar 2016a	62	135.43	24.80	17	146.35	21.30	-10.92 [-23.86, 2.02]	19.57
Kumari 2018	50	122.76	22.66	25	151.60	16.18	-28.84 [-38.80, -18.88]	22.97
Heterogeneity: $\tau^2 = 125.85$, $I^2 = 78.38\%$, $H^2 = 4.63$							-20.38 [-37.91, -2.84]	
Test of $\theta_i = \theta_j$: Q(1) = 4.63, p = 0.03								
**Others**								
Dans 2007	20	143.95	13.87	20	151.33	72.24	-7.38 [-39.62, 24.86]	6.77
Trakoon-osot 2013	19	-7.11	13.92	19	-2.84	10.78	-4.27 [-12.19, 3.65]	25.33
Cortez-Navarrete 2018	10	133.32	52.25	10	129.72	41.44	3.60 [-37.73, 44.93]	4.50
Amini 2020	9	168.78	38.24	9	189.00	37.22	-20.22 [-55.08, 14.64]	5.98
Kim 2020	62	140.50	31.90	28	155.10	53.00	-14.60 [-32.27, 3.07]	14.89
Heterogeneity: $\tau^2 = 0.00$, $I^2 = 0.00\%$, $H^2 = 1.00$							-6.34 [-13.16, 0.47]	
Test of $\theta_i = \theta_j$: Q(4) = 1.94, p = 0.75								
**Overall**							-13.56 [-23.06, -4.07]	
Heterogeneity: $\tau^2 = 76.37$, $I^2 = 56.74\%$, $H^2 = 2.31$								
Test of $\theta_i = \theta_j$: Q(6) = 15.41, p = 0.02								
Test of group differences: $Q_b(1) = 2.14$, p = 0.14								

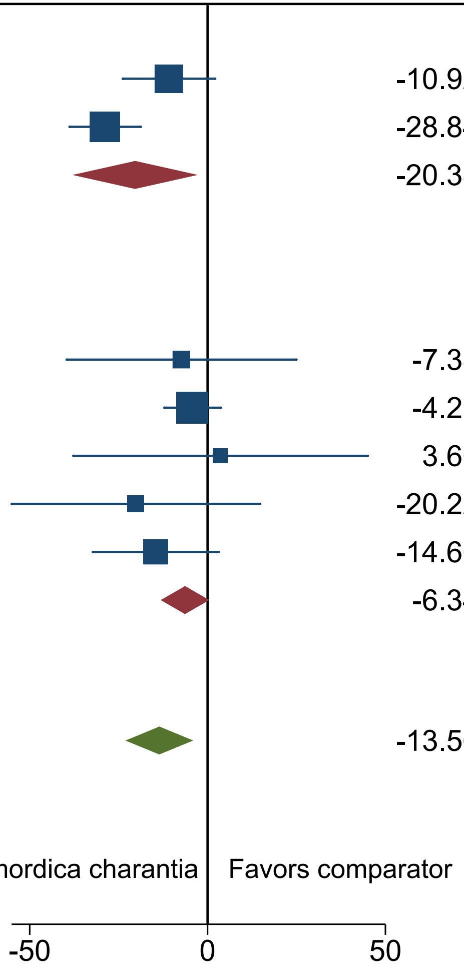

Random-effects REML model
